# Characterization of the Volatile Compounds of the Hardwood Portion of *Betula papyrifera* Marshall From Quebec, Canada

**DOI:** 10.1002/cbdv.202501398

**Published:** 2025-07-18

**Authors:** David Fortier, Jean‐Christophe Séguin, Normand Voyer

**Affiliations:** ^1^ Département De Chimie PROTEO and Centre d'études nordiques Université Laval Québec Canada

**Keywords:** *Betula papyrifera*, fatty acids, hardwood composition, natural products, volatile extract

## Abstract

*Betula papyrifera* Marshall (paper birch) hardwood is an abundant yet underutilized resource for Quebec's forestry industry. We investigated the volatile compounds of the hardwood extracted using hydrodistillation (HD) and headspace solid‐phase microextraction (HS‐SPME) and analyzed them by gas chromatography–mass spectrometry (GC–MS) and GC‐flame ionization detection. HD produced an essential oil with a low average yield (0.010% ± 0.001%), from which we identified 51 compounds, dominated by linoleic acid and its oxidation products. HS‐SPME provided a complementary profile, with 50 compounds identified, including aromatics and sesquiterpenes absent from the essential oil. The findings suggest that direct valorization of *B. papyrifera* hardwood for its volatile secondary metabolites is limited due to low yields and the prevalence of common compounds. Nevertheless, the study provides novel insights into the volatile chemical composition of *B. papyrifera*, contributing to the fundamental understanding of its extractives profile.

## Graphical abstract

The volatile profile of Betula papyrifera hardwood has been characterized using both hydrodistillation and HS‐SPME coupled with GC‐MS/FID. Hydrodistillation yields a fatty acid‐rich essential oil, while HS‐SPME reveals a broader range of low‐molecular‐weight volatiles, including aromatics and terpenoids. While findings highlight limited potential for direct valorization, it provides new insights into an underutilized forest resource.

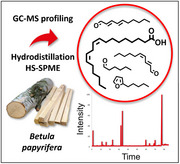

## 1 Introduction

The paper birch or white birch, scientifically known as *Betula papyrifera* Marshall, is easily identifiable by its distinctive white bark, which gradually exfoliates into thin lateral strips over time [1]. The peeling bark is the main characteristic that sets it apart from poplars and yellow birch, whose bark does not peel off. Birch's white bark contains lenticels, identifiable by horizontal black streaks, and is embellished with black whisker‐like markings along the trunk. It is often found in mixed forests but also sometimes as a pioneer after fires or tree removal. *B. papyrifera* is distributed across North America and is particularly abundant in Canada and the northern United States. Geographically, it ranges from Alaska in the west to Newfoundland and Labrador in the east, and from throughout all southern regions of Canada to high latitudes in the north [1]. In Quebec, birch grows at least as far north as 55° N, with anecdotal observations suggesting occurrences as far north as 58° N [2]. Birch thrives in well‐drained, sandy, and cold soils. Upon reaching maturity, the tree typically attains a height ranging from 15 to 21 m, with an average diameter spanning 0.3–0.6 m [3, 4]. Throughout history, indigenous communities in North America have utilized the tree's bark to craft items such as canoes, baskets, and food containers [5].

Although the *B. papyrifera* is already an abundant species in Quebec, recent observations have revealed significant population changes. For example, in 2011 in the region of Saguenay Lac‐Saint‐Jean, a study noted that coniferous populations were decreasing in favor of deciduous trees [6]. This shift has resulted in a predominant presence of *B. papyrifera* and trembling aspen, *Populus tremuloides* Michx, in the region. Unfortunately, these two species, often undervalued by the forest industry, are frequently cut down but left unused on logging sites, representing a significant waste [7]. The surge in these tree species gives rise to multiple concerns as it creates changes in plant composition of ecosystems, thus creating challenges in terms of biodiversity and forest economics. In 2023, Canadian forests witnessed an unprecedented surge in wildfires, covering approximately 15 million hectares, representing about 4% of the total forest area, and greater than seven times the average annual burned area recorded over the past 40 years [8]. These fires continued to engulf large areas of the country in 2024 [9], further compounding their environmental impact. This raises concerns about the *B. papyrifera*’s tendency to thrive as a pioneer species after wildfires [10]. This highlights the importance of developing new strategies to better integrate *B. papyrifera* into the forestry industry. Doing so would help justify the challenges related to its exploitation and prevent the underutilization of this abundant and expanding forest resource.

New and innovative ways are being developed to add value to forest residues. A number of valorization projects have already highlighted potential new uses [11]. There are numerous studies on extractables from *B. papyrifera*, almost exclusively focused on the bark, which is particularly rich in pentacyclic triterpenes, interesting for their bioactive properties. Betulin, a common pentacyclic triterpene, is believed to be present in more than 200 plants and is responsible for the characteristic white color of the bark of *B. papyrifera*, in which it is particularly abundant [12, 13]. The bark mainly contains betulin (20%–30%, or even up to 45%) that can easily be purified from the mixture by recrystallization [14, 15]. The bark also contains, to a lesser extent but in significant proportions, lupeol, betulinic acid, and betulin 3‐caffeate. Other betulin‐derived triterpenoids, such as betulinic aldehyde, oleanolic acid, oleanolic acid‐3‐acetate, and other compounds, have been identified in various species of the genus *Betula* [16]. Betulin and its numerous natural and synthetic derivatives are particularly bioactive and have been studied, among other things, for their antiviral, antitumor, antibacterial, anti‐inflammatory, and anti‐HIV properties, and have also been the subject of studies to evaluate their potential in the treatment of metabolic disorders, infectious diseases, and cardiovascular and neurological disorders [12, 13, 16, 17, 18, 19].

Although *B. papyrifera* extractive compounds have been the focus of increased research interest over the past decade, the volatile compounds in its hardwood remain mostly unexplored, presenting new opportunities for investigation into its unused potential. Wood from other trees, such as sandalwood (*Santalum album* L.), camphorwood (*Cinnamomum camphora* L.), or rosewood (*Aniba duckei* Ducke), are economically important sources of interesting volatile compounds known as extractives [11]. Moreover, solvent extractives of the wood from other birch species led to interesting lipophilic extracts containing volatile compounds such as those that can be extracted by processes like hydrodistillation (HD) [20, 21]. The hardwood of trees contains many organic compounds: Polysaccharides, such as cellulose, hemicellulose, and lignin, give the wood its distinct structural properties, whereas other extractive compounds are found in much smaller quantities. Within the intricate physiology of plants, extractives have three distinct roles: serving as nutrient reserves, acting as plant hormones, and protecting the wood [22]. They contribute to many characteristic properties of wood, such as its odor, color, toxicity, stability to light, flammability, hygroscopicity, degradation, insect resistance, and permeability [23]. These compounds, often of low molecular weight, encompass different classes, including lipophilic compounds like fats, fatty acids, sterols, terpenoids, terpenes, and fatty alcohols or hydrosoluble compounds such as polyphenols, sugars, and lignans [24]. The organic compound composition in wood exhibits variability influenced by factors such as genetics, season, and age and significantly differs depending on the tree species.

HD is a traditional method of volatile compound extraction that has been employed for centuries and maintains economic significance, especially in the perfume and aromatherapy industries [25, 26, 27]. The extraction of volatile compounds by HD does not alter the wood other than dampening it; the wood could still be used in other valuable ways after extraction. Headspace solid‐phase microextraction (HS‐SPME) is a highly efficient and non‐destructive method for collecting volatile compounds while minimizing compound degradation, thus allowing analyzed volatilome to be closer to the real composition of the volatile compounds produced by the organism itself [28, 29]. Although being an interesting extraction method for volatilome characterization, it is not suited for large‐scale recovery of extractives for industrial applications and is used as a complementary scientific investigation technique. Given that HD is more sensitive to high molecular weight compounds and favors degradation, the two methods are often viewed as complementary extraction methods for the analysis of volatile compounds [30].

Herein, we report the detailed characterization of the volatile profile of the hardwood of *B. papyrifera*. Two extraction methods were employed: HD, yielding the essential oil, and HS‐SPME. The overarching goals include acquiring fundamental knowledge about the volatilome of *B. papyrifera* hardwood, identifying commercially valuable or scientifically interesting molecules, and finding potential applications for *B. papyrifera* for the forestry industry.

## 2 Results and Discussion

### 2.1 Essential Oil Yields

Three trees were sampled in total, and three essential oils were obtained per tree from fresh wood using a 5‐h HD process. The extract obtained from birch was colorless to pale yellow, with an aroma reminiscent of grilled chicken and freshly cut green leaves. Previous studies on *Betula pubescens* Ehrh. and *B. pendula* Roth showed that the upper part of the tree may be richer in extractive compounds [31]. Therefore, to obtain a more representative extract of the entire tree, we selected pieces from the bottom, middle, and upper sections, blended them together, and performed three distillations on the resulting mix. These were then used to calculate the mean and standard deviation. The ages of the trees selected for the study were 54, 36, and 83 years. The youngest tree (Tree 2) exhibited a slightly lower volatile extract yield (calculated from dry mass) compared to the 2 older trees. They exhibited an average of triplicate yields (% w/w on dry mass) of 0.0114% ± 0.0008%, 0.0087% ± 0.0006%, and 0.010% ± 0.001% for Trees 1, 2, and 3, respectively. These yields can be considered particularly low, as HD of the same quantity of *S. album* L. hardwood (sandalwood) by Bisht et al. [32] resulted in yields of 1.6%–3.6%, more than 300 times higher than those obtained from *B. papyrifera*. Lower yields of volatile compounds are generally expected from the wood than other parts of the tree. For instance, Roitto et al. [31] observed that extractives from *B. pendula* and *B. pubescens* were almost double in branch biomass and 5–10 fold in bark than the from the stemwood.

Although the yield is low, extracting phenols from wood by hot‐water extraction could easily be combined with the recovery of an essential oil with the same process. Indeed, other research projects on the hot‐water extraction of wood phenols have demonstrated that it is an interesting way to valorize industrial woodwork residues [33]. It may be possible to improve the low yields obtained and add more value to *B. papyrifera* wood by HD.

### 2.2 Essential Oils Composition

**FIGURE 1:**
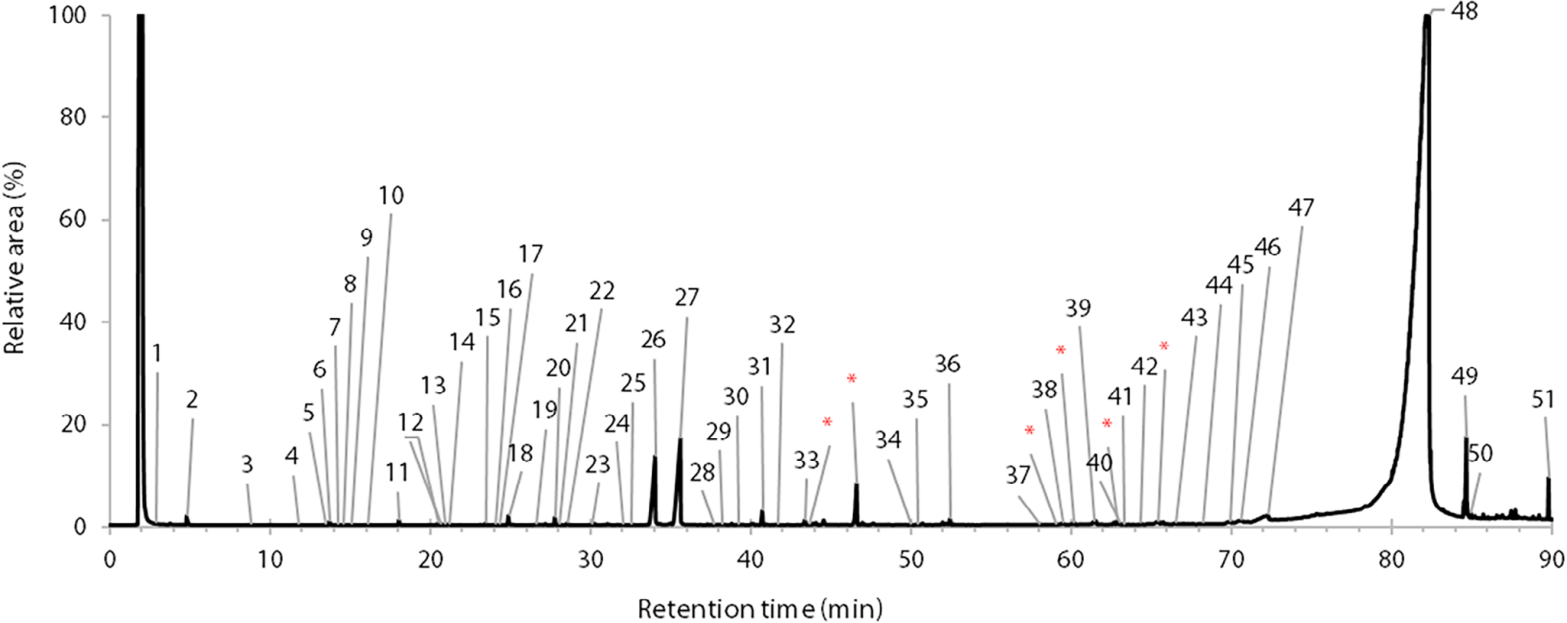
Typical chromatogram of the essential oils of the hardwood of *Betula papyrifera* from St‐Julien, Québec, Canada. The labels on the signals correspond to the compound number in Table 1. Asterisks represent artifacts, including butylated hydroxytoluene and derivatives, detected in the blank and excluded from the integration process.

A total of 51 compounds, constituting 96.6% (Tree 2) and 97.6% (Trees 1 and 3) of the total integrated area, were identified in the essential oils of *B. papyrifera*. A typical chromatogram of the volatile compounds in *B. papyrifera* is shown in Figure 1, and the peak assignments are presented in Table 1. Other chromatograms were very similar and are presented in the Supporting Information section. The compounds were separated and identified using gas chromatography–mass spectrometry (GC–MS), with semi‐quantitative proportions determined via GC‐flame ionization detection (FID).

**TABLE 1:** Volatile organic compounds (VOCs) identified in the essential oils of the three *B. papyrifera* trees using mass spectrometry and linear retention index comparisons.

No.	LRI		Compound name	No. CAS	Relative area (% ± SD)^a^		
	Exp.^b^	Lit.			Tree 1	Tree 2	Tree 3
1	703	700^c^	Heptane	142‐82‐5	Tr	Tr	Tr
2	802	800^c^	Hexanal	66‐25‐1	0.1 ± 0.0	0.1 ± 0.0	Tr
3	902	906^d^	Heptanal	111‐71‐7	Tr	Tr	Tr
4	954	958 or 956^c^	(*E* or *Z*)‐2‐heptenal	57266‐86‐1 or 18829‐55‐5	Tr	Tr	Tr
5	985	986^c^	6‐Methyl‐5‐hepten‐2‐one	110‐93‐0	Tr	Tr	Tr
6	989	991^d^	2‐Pentylfuran	3777‐69‐3	0.1 ± 0.0	0.1 ± 0.0	0.1 ± 0.0
7	998	1002^c^	(*E* or *Z*)‐2‐(2‐pentenyl)furan	70424‐14‐5 or 70424‐13‐4	Tr	Tr	Tr
8	1003	1003^c^	Octanal	124‐13‐0	Tr	Tr	Tr
9	1010	1013^d^	(*E,E*)‐2,4‐heptadienal	881395	Tr	Tr	Tr
10	1026	1031^c^	3‐Ethyl‐2‐methyl‐1,3‐hexadiene	61142‐36‐7	Tr	Tr	Tr
11	1055	1059^d^	(*E*)‐2‐Octenal	2363‐89‐5	0.1 ± 0.0	0.1 ± 0.1	0.1 ± 0.0
12	1095	1105^c^	(*E*)‐4‐Nonenal	2277‐16‐9	Tr	Tr	Tr
13	1099	1101^d^	Linalool	78‐70‐6	Tr	Tr	Tr
14	1103	1107^d^	Nonanal	124‐19‐6	Tr	Tr	Tr
15	1136	1134^d^	Non‐3‐en‐2‐one	14309‐57‐0	Tr	Tr	Tr
16	1144	1148^c^	(*Z*)‐2‐nonenal	60784‐31‐8	Tr	Tr	Tr
17	1149	1155 or 1152^c^	(*E,E*) or (*E,Z*)‐2,6‐nonadienal	17587‐33‐6 or 557‐48‐2	Tr	Tr	Tr
18	1157	1163^d^	(*E*)‐2‐nonenal	18829‐56‐6	0.2 ± 0.0	0.1 Tr 0.0	0.1 ± 0.0
19	1182	1192^d^	Octanoic acid	124‐07‐2	Tr	Tr	Tr
20	1200	1200^c^	Dodecane	112‐40‐3	0.1 ± 0.0	0.2 ± 0.1	0.1 ± 0.0
21	1204	1208^d^	Decanal	112‐31‐2	Tr	Tr	Tr
22	1211	1218^d^	(*E,E*)‐2,4‐nonadienal	5910‐87‐2	Tr	Tr	Tr
23	1234	1238^d^	Neral	106‐26‐3	Tr	Tr	Tr
24	1264	1268^d^	Geranial	141‐27‐5	Tr	Tr	Tr
25	1272	1273^c^	Nonanoic acid	112‐05‐0	Tr	Tr	Tr
**26**	**1294**	**1292** ^c^	**(*E,Z*)‐2,4‐decadienal**	**25152‐83‐4**	**2.2** ± **0.3**	**2.8** ± **0.2**	**1.1** ± **0.2**
**27**	**1318**	**1322** ^d^	**(*E,E*)‐2,4‐decadienal**	**25152‐84‐5**	**3.3** ± **0.3**	**4.3** ± **0.1**	**1.9** ± **0.3**
28	1353	1363^c^	γ‐Nonalactone	104‐61‐0	Tr	Tr	Tr
29	1361	1365^d^	(*Z*)‐8‐undecenal	147159‐49‐7	Tr	Tr	Tr
30	1375	1386^d^	9‐Decenoic acid	14436‐32‐9	—	Tr	Tr
31	1400	1400^c^	Tetradecane	629‐59‐4	0.1 ± 0.1	0.3 ± 0.1	0.2 ± 0.0
32	1416	1425^d^	(*E,E*)‐2,4‐undecadienal	30361‐29‐6	Tr	Tr	Tr
33	1444	1453^c^	(*E*)‐geranyl acetone	3796‐70‐1	0.1 ± 0.0	0.1 ± 0.0	0.1 ± 0.0
34	1557	1561^d^	(*E*)‐nerolidol	40716‐66‐3	Tr	Tr	Tr
35	1563	1568^c^	Dodecanoic acid	143‐07‐7	Tr	Tr	Tr
36	1599	1600^d^	Hexadecane	544‐76‐3	0.1 ± 0.0	0.1 ± 0.1	0.1 ± 0.0
37	1702	1714^d^	(*E,Z*)‐2,6‐farnesal	3790‐67‐8	Tr	Tr	Tr
38	1730	1735^c^	(*E,E*)‐2,6‐farnesal	502‐67‐0	Tr	Tr	Tr
39	1767	1768^c^	Tetradecanoic acid	544‐63‐8	0.1 ± 0.1	0.1 ± 0.0	0.1 ± 0.0
40	1799	1800^d^	Octadecane	593‐45‐3	Tr	Tr	Tr
41	1804	1818^d^	(*E,E*)‐2,6‐farnesoic acid	462‐11‐3	Tr	Tr	Tr
42	1825	1832^d^	Farnesyl acetate	29548‐30‐9	Tr	Tr	Tr
43	1866	1867^c^	Pentadecanoic acid	1002‐84‐2	Tr	—	—
44	1903	—	Farnesyl acetone isomer	—	Tr	Tr	Tr
45	1935	1929^d^	Ambrettolide	123‐69‐3	0.2 ± 0.1	—	—
46	1944	1951^c^	Palmitoleic acid	373‐49‐9	0.2 ± 0.1	0.1 ± 0.0	Tr
**47**	**1977**	**1968** ^c^	**Hexadecanoic acid**	**57‐10‐3**	**1.3** ± **0.9**	**0.2** ± **0.1**	**0.1** ± **0.0**
**48**	**2126**	**2133** ^c^	**Linoleic acid**	**60‐33‐3**	**87.7** ± **1.4**	**85.0** ± **1.3**	**91.8** ± **0.6**
**49**	**2375**	**2386** ^c^	**(*Z*)‐9‐octadecenamide**	**301‐02‐0**	**1.2** ± **0.2**	**1.8** ± **0.3**	**1.3** ± **0.5**
50	2391	2374^c^	Octadecanamide	124‐26‐5	0.1 ± 0.0	0.1 ± 0.0	0.1 ± 0.0
51	2815	2832^c^	Squalene	111‐02‐4	0.4 ± 0.0	0.8 ± 0.2	0.3 ± 0.1

*Note*: **In bold,** the main compounds (>1%).

^a^ Relative areas are calculated based on the average of nine replicates (three analyses from three separate distillations) for each of the three trees (1, 2, and 3); tr: relative proportion is marked as trace for values <0.1%.

^b^ Database linear retention indices (LRIs) shown in this table for non‐polar column DB‐5MS are obtained according to standards of *n*‐alkanes (C7–C30).

^c^LRI in NIST 14 database Mainlib and Replib [34].

^d^LRI in FFNSC 3 database [35].

*B. papyrifera* hardwood essential oil is rich in unsaturated fatty acids and their oxidation derivatives. Its profile is largely dominated by linoleic acid (85.0% ± 1.3% to 91.8% ± 0.6%), a fatty acid common to many plants and organisms [36, 37]. Other major components include (*E*,*E*)‐2,4‐decadienal (1.9% ± 0.3% to 4.3% ± 0.1%), (*E,Z*)‐2,4‐decadienal (1.1% ± 0.2% to 2.8% ± 0.2%), (*Z*)‐9‐octadecenamide (1.2% ± 0.2% to 1.8% ± 0.3%), hexadecanoic acid (0.1% ± 0.0% to 1.3% ± 0.9%), squalene (0.3% ± 0.1% to 0.8% ± 0.2%), tetradecane (0.1% ± 0.1% to 0.3% ± 0.1%), and ambrettolide, identified only in Tree 1 (0.2% ± 0.1%).

Among the identified compounds, aldehydes constitute the most prevalent class in the essential oil, with 21 compounds, of which only 5 are saturated, namely, hexanal, heptanal, octanal, nonanal, and decanal. The remaining 16 are unsaturated compounds, including lipid oxidation products and terpenoids. Among these, the two most abundant compounds in the extract, following linoleic acid, are the isomers (*E,Z*)‐2,4‐decadienal and (*E,E*)‐2,4‐decadienal. Specimens with higher linoleic acid content exhibit lower proportions of these isomers, and vice versa. These 2 isomers, along with hexanal, are recognized as primary oxidation products of linoleic acid [38]. In the food industry, it has been observed that when using linoleic acid‐rich oils for frying, the concentrations of 2,4‐decadienal isomers increase progressively over time [39]. These compounds are considered significant contributors to the aroma of grilled chicken and frying in general [38, 40], which may account for the distinctive scent of the essential oil.

Carboxylic acids, specifically fatty acids in this context, represent the second most identified class of compounds in the essential oil, with nine compounds detected. They are the most significant in terms of proportion, largely due to the high abundance of linoleic acid. Fatty acids originate from the saponification of structural lipids constituting the organism [27]. Hexadecanoic acid, more commonly known as palmitic acid, was identified in varying proportions. Although considerably less abundant, it is the second‐most‐abundant fatty acid in the essential oil of *B. papyrifera*, after linoleic acid. Palmitic acid is the most common saturated fatty acid in the human body and is very common in many animals and plants [41]. Trace amounts of other linear saturated fatty acids were identified, including pentadecanoic, tetradecanoic, dodecanoic, nonanoic, and octanoic. Additionally, two unsaturated fatty acids were observed: 9‐decenoic and (*E,E*)‐2,6‐farnesoic.

The two nitrogen compounds identified, (*Z*)‐9‐octadecenamide and octadecanamide, originate most probably from the cleavage of sphingolipids, which are lipids composed of a sphingosine on which a fatty acid is amidated, in this case oleic and stearic acid [42]. Sphingolipids are essential components of plasma membranes and other endomembranes of plant cells [43]. The two compounds identified are primary amides. (*Z*)‐9‐octadecenamide was identified in a particularly high relative proportion, varying on average between 1.2% ± 0.2% and 1.8% ± 0.3%, whereas octadecanamide was in trace amounts.

Terpenes and terpenoids are generally important for the valorization of essential oils due to their diverse bioactive properties and odorous profiles [26], making their identification and analysis pertinent for understanding the extract's potential applications. Three monoterpenoids were identified in small proportions: linalool, neral, and geranial. Neral and geranial, both isomers of citral found in many flower and herb species, have a pleasant citrus aroma and are oxidation derivatives of geraniol [44]. Linalool, with its fresh, flowery scent, is a component of numerous essential oils and is widely used in the fragrance industry [45].

All the identified sesquiterpenoids were identified in trace amounts and belong to the farnesene family, including compounds such as (*E*)‐nerolidol, (*E,E*)‐ and (*E,Z*)‐2,6‐farnesal, (*E,E*)‐farnesoic acid, farnesyl acetate, and an unidentified isomer of farnesyl acetone (Compound **44**).

These sesquiterpenoids are likely derived from farnesyl pyrophosphate (FPP), which, after cleavage from the terminal phosphate group by a phosphatase, can lead to the formation of various derivatives [46]. FPP can undergo hydrolysis to form 2,6‐(*E,E*)‐farnesol or (*E*)‐nerolidol. The latter, a sesquiterpenoid with a woody odor reminiscent of fresh bark, is found in many plants such as ginger, jasmine, lavender, lemongrass, and cannabis [47]. Farnesol can then be oxidized to produce aldehydes or carboxylic acids, leading to the formation of (*E,E*)‐2,6‐farnesal and (*E,E*)‐2,6‐farnesoic acid. The presence of an unsaturation in the trans configuration of FPP explains why most of the metabolites observed in this study exhibit the same configuration. Given that HD creates oxidative conditions, it is challenging to confirm whether some of these metabolites were formed during the extraction process or whether they are naturally produced by the plant itself.

Squalene, a triterpene, was identified as the molecule with the highest molecular weight in the essential oil, present in proportions ranging from 0.3% ± 0.1% to 0.8% ± 0.2%. Squalene is known to be a precursor for the biosynthesis of other triterpenes, including betulin and betulinic acid, which are major components of birch bark [16, 48]. Neither of these compounds was detected in the essential oil. Squalene has also been identified as a major compound in *B. papyrifera* wood extracts obtained via Soxhlet extraction by Lavoie and Stevanovic [49], where it was found at a concentration of 4.062 mg/g (dry mass).

### 2.3 HS‐SPME/GC–MS

**FIGURE 2:**
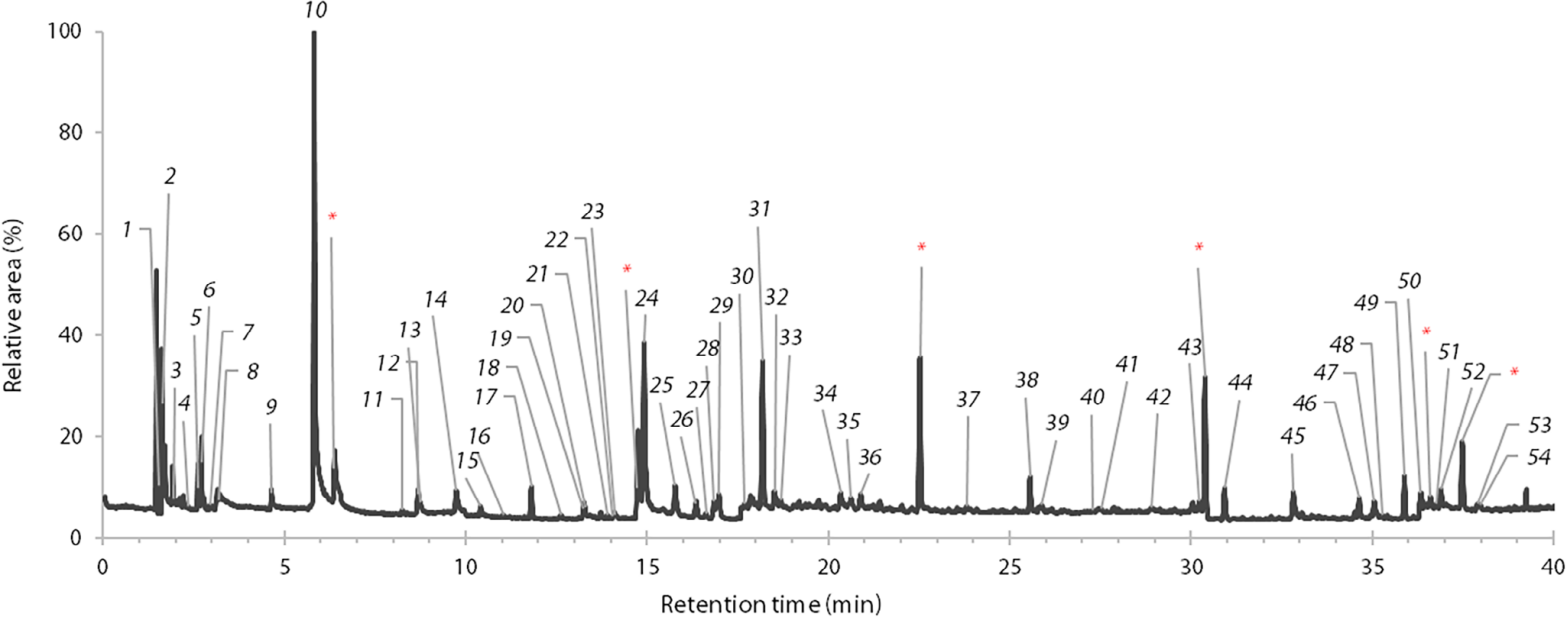
Typical HS‐SPME/GC–MS chromatogram of the hardwood of *Betula papyrifera* from St‐Julien, Québec, Canada. The labels on the signals correspond to the compound number in Table 2. Asterisks represent siloxanes that were also detected in the blank and ignored in the integration process.

Wood chips from the hardwood of three freshly cut *B. papyrifera* trees were analyzed in triplicate by HS‐SPME–GC–MS. The typical chromatogram obtained is shown in Figure 2. The chromatograms of the other replicates are available in the Supporting Information section. A total of 50 compounds were identified in the headspace and are presented in Table 2, accounting for 80.8%, 75.1%, and 71.7% of the area of compounds integrated on the chromatogram for Trees 1, 2, and 3, respectively. The high variation in the percentage of identified compounds is due largely to the high variability of unidentified Compound **31** (6%–20%). The chromatogram of the headspace portion showed many signals, sometimes overlapping or with very low intensity. A significant portion of the identified compounds were unsaturated or branched, predominantly alkenes and sesquiterpenes. This made the identification process more difficult, as databases provided either too many possible molecules or no satisfactory options. Due to these factors, the percentages of identified compounds are lower using HS‐SPME, especially in the absence of linoleic acid. Linoleic acid constitutes a significant portion of the essential oil composition but is completely absent from the headspace analysis. This is most probably due to its high molecular weight and low volatility at the temperature used for headspace sampling.

**TABLE 2:** Volatile organic compounds (VOCs) identified in the headspace of 3 *Betula papyrifera* trees.

No.	LRI		Compound name	No. CAS	Relative area (% ± SD)^a^		
	Exp.^b^	Lit.			Tree 1	Tree 2	Tree 3
1	<600	401^c^	Acetaldehyde^e^	75‐07‐0	2.4 ± 1.2	1.8 ± 0.1	1.2 ± 0.4
**2**	**<600**	**427** ^c^	**Ethanol** ^e^	**64‐17‐5**	**3.7** ± **1.2**	**2.4** ± **0.2**	**0.5** ± **0.1**
3	<600	552^c^	2‐Methylpropanal^e^	78‐84‐2	0.9 ± 0.1	0.9 ± 0.1	0.8 ± 0.1
4	605	631^c^	Methylcyclopentane	96‐37‐7	tr	Tr	0.1 ± 0.0
5	636	676^d^	3‐Methylbutanal	590‐86‐3	1.7 ± 0.6	1.4 ± 0.2	1.5 ± 0.2
**6**	**648**	**662** ^c^	**2‐Methylbutanal**	**96‐17‐3**	**2.4** ± **0.7**	**1.8** ± **0.1**	**2.2** ± **0.3**
7	673	680^d^	1‐Penten‐3‐ol	616‐25‐1	0.3 ± 0.1	0.2 ± 0.0	0.1 ± 0.1
8	695	699^c^	Pentanal	110‐62‐3	0.4 ± 0.1	0.4 ± 0.1	0.7 ± 0.2
9	756	763^c^	Toluene	108‐88‐3	1.5 ± 1.3	0.8 ± 0.2	0.3 ± 0.2
**10**	**801**	**800** ^c^	**Hexanal**	**66‐25‐1**	**26.1** ± **2.7**	**25.4** ± **3.4**	**27.4** ± **4.2**
11	854	855^c^	Ethylbenzene	100‐41‐4	0.2 ± 0.0	0.2 ± 0.1	0.2 ± 0.0
12	864	865^c^	*p*‐Xylene	106‐42‐3	1.1 ± 0.1	0.8 ± 0.3	0.5 ± 0.1
13	866	866^c^	*m*‐Xylene	108‐38‐3	0.8 ± 0.4	0.4 ± 0.1	0.4 ± 0.1
14	888	887^c^	*o*‐Xylene	95‐47‐6	1.7 ± 0.2	1.2 ± 0.3	0.9 ± 0.3
15	902	901^c^	Heptanal	111‐71‐7	0.5 ± 0.1	0.4 ± 0.1	0.7 ± 0.1
16	914	920^c^	Anisole	100‐66‐3	0.1 ± 0.0	0.4 ± 0.1	1.2 ± 0.2
17	929	929^c^	α‐Pinene	80‐56‐8	1.6 ± 0.3	0.8 ± 0.1	3.0 ± 0.3
18	944	952^c^ or 950^c^	Camphene or α‐fenchene	79‐92‐5 or 471‐84‐1	0.3 ± 0.1	0.2 ± 0.1	0.8 ± 0.1
19	956	958^c^ or 956^c^	(*E* or *Z*)‐2‐Heptenal	57266‐86‐1 or 18829‐55‐5	0.1 ± 0.1	0.2 ± 0.1	0.1 ± 0.1
20	957	957^c^ or 954^c^	(*m* or *p*)‐Methyltoluene	620‐14‐4 or 622‐96‐8	1.0 ± 0.2	0.6 ± 0.3	0.7 ± 0.1
21	969	974^c^ or 966^c^	Sabinene or β‐thujene	3387‐41‐5 or 28634‐89‐1	0.1 ± 0.0	0.2 ± 0.1	0.7 ± 0.0
22	973	979^c^ or 974^c^	β‐Pinene or sabinene	127‐91‐3 or 3387‐41‐5	0.5 ± 0.0	0.3 ± 0.1	1.1 ± 0.0
23	973	970^c^	*o*‐Ethyltoluene	611‐14‐3	Tr	Tr	Tr
**24**	**988**	**991** ^d^	**2‐Pentylfuran**	**3777‐69‐3**	**10.8** ± **1.3**	**7.0** ± **1.0**	**3.1** ± **0.9**
**25**	**1005**	**1009** ^d^	***o*‐Methylanisole**	**578‐58‐5**	**1.8** ± **0.1**	**2.8** ± **0.3**	**2.1** ± **0.2**
26	1016	1013^c^	1,2,3‐Trimethylbenzene	526‐73‐8	1.1 ± 0.2	0.6 ± 0.2	0.9 ± 0.4
27	1021	1025^c^	*p*‐Cymene	99‐87‐6	0.2 ± 0.0	0.4 ± 0.2	0.5 ± 0.1
28	1025	1030^d^	d‐Limonene	138‐86‐3	1.0 ± 0.2	1.3 ± 0.2	1.3 ± 0.2
**29**	**1028**	**1032** ^d^	**Eucalyptol**	**470‐82‐6**	**1.5** ± **0.4**	**1.9** ± **0.4**	**4.0** ± **0.5**
30	1042	1045^c^	Phenylacetaldehyde	122‐78‐1	0.3 ± 0.2	Tr	0.3 ± 0.1
**31**	**1052**	**—**	**Unknown** ^f^	**—**	**6.0** ± **1.9**	**6.7** ± **1.5**	**19.7** ± **4.3**
32	1058	1059^d^	(*E*)‐2‐Octenal	2363‐89‐5	0.9 ± 0.3	0.8 ± 0.3	0.7 ± 0.3
33	1061	1058^c^	2,6,7‐Trimethyldecane	62108‐25‐2	0.4 ± 0.1	0.6 ± 0.0	0.5 ± 0.1
34	1094	1105^c^	(*E*)‐4‐Nonenal	2277‐16‐9	0.7 ± 0.1	0.4 ± 0.1	0.4 ± 0.2
35	1099	1100^c^	Undecane	1120‐21‐4	0.5 ± 0.2	0.4 ± 0.1	0.4 ± 0.1
36	1104	1104^c^	Nonanal	124‐19‐6	1.0 ± 0.2	0.6 ± 0.1	0.8 ± 0.3
37	1164	1164^c^	2‐Methylundecane	7045‐71‐8	0.2 ± 0.0	0.3 ± 0.1	0.2 ± 0.1
38	1199	1200^d^	Dodecane	112‐40‐3	1.7 ± 0.3	1.2 ± 0.2	0.4 ± 0.0
39	1206	1206^c^	Decanal	112‐31‐2	0.3 ± 0.1	0.4 ± 0.1	0.2 ± 0.0
40	1237	1238^c^	Hexylcyclohexane	4292‐75‐5	0.1 ± 0.1	0.2 ± 0.0	0.1 ± 0.0
41	1242	1242^c^	4‐Ethylundecane	17312‐59‐3	0.4 ± 0.2	0.3 ± 0.1	0.2 ± 0.1
42	1271	1289^d^	Acide nonanoïque	112‐05‐0	0.7 ± 0.4	0.5 ± 0.3	0.6 ± 0.1
43	1300	1300^c^	Tridecane	629‐50‐5	0.5 ± 0.1	0.4 ± 0.2	0.2 ± 0.1
**44**	**1315**	**—**	**Unknown** ^f^	**—**	**2.4** ± **0.7**	**3.6** ± **2.1**	**1.2** ± **0.3**
45	1359	1363^c^	γ‐Nonalactone	104‐61‐0	1.9 ± 0.2	0.5 ± 0.2	0.2 ± 0.1
46	1400	1400^d^	Tetradecane	629‐59‐4	1.1 ± 0.6	1.8 ± 0.4	0.6 ± 0.0
**47**	**1415**	**1416** ^d^	**(*Z*)‐α‐Bergamotene**	**18252‐46‐5**	**1.2** ± **0.2**	**2.0** ± **0.2**	**1.3** ± **0.3**
48	1422	1418^d^	α‐Santalene	512‐61‐8	0.3 ± 0.2	0.3 ± 0.1	0.5 ± 0.2
**49**	**1444**	**1435** ^c^	**(*E*)‐α‐Bergamotene**	**13474‐59‐4**	**2.1** ± **0.2**	**4.8** ± **0.7**	**3.6** ± **0.7**
50	1459	**—**	C15H24 (Sesquiterpene)^f^	**—**	0.8 ± 0.2	1.5 ± 0.2	0.8 ± 0.1
51	1474	**—**	C15H24 (Sesquiterpene)^f^	—	0.3 ± 0.3	1.1 ± 0.1	0.3 ± 0.1
**52**	**1479**	**1483** ^d^	**(*E*)‐β‐Bergamotene**	**15438‐94‐5**	**0.7** ± **0.5**	**2.6** ± **0.3**	**1.2** ± **0.1**
53	1512	1505^c^	Cuparene	16982‐00‐6	0.3 ± 0.1	0.5 ± 0.1	0.3 ± 0.1
54	1534	1524^c^	β‐Sesquiphellandrene	20307‐83‐9	0.3 ± 0.0	0.6 ± 0.2	0.3 ± 0.1

*Note*: **In bold,** the main compounds (>2%).

Abbreviation: LRIs, linear retention indices.

^a^ Relative areas are calculated based on the average of three replicates for each of the three trees; tr: relative proportion is marked as trace for values <0.1%.

^b^ Database linear retention indices shown in this table for non‐polar column DB‐5MS are obtained according to standards of *n*‐alkanes (C6‐C30).

^c^ LRI in NIST 14 database Mainlib and Replib [34].

^d^ LRI in FFNSC 3 database [35].

^e^ Tentative identification made solely from mass spectrum evaluation.

^f^ Several possible isomers, mass spectra:[No.31] 41 (15), 42 (16), 43 (23), 55 (21), 56 (14), 84 (88), 86 (21), 87 (100), 97 (28), 112 (24); [No.44] 41 (18), 43 (17), 55 (11), 56 (66), 57 (100), 69 (7), 71 (20), 85 (11), 140 (11), 183 (6); [No.50] 41 (77), 55 (46), 69 (100), 79 (66), 91 (53), 93 (86), 105 (40), 107 (40), 119 (72), 161 (54); [No.51] 41 (38), 44 (31), 55 (31), 69 (100), 79 (34), 91 (42), 93 (62), 105 (30), 120 (37), 161 (47).

The two main volatile compounds obtained by HS‐SPME are hexanal (25.4% ± 3.4% to 27.4% ± 4.2%) and 2‐pentylfuran (3.1% ± 0.9% to 10.8 ± 1.3%), both also identified in the essential oil and derived from linoleic acid. Only 11 compounds were identified in both extracts obtained by the two extraction methods: nonanoic acid, hexanal, heptanal, an isomer of 2‐heptenal, 2‐pentylfuran, (*E*)‐2‐octenal, nonanal, (*E*)‐4‐nonenal, dodecane, decanal, nonanoic acid, and γ‐nonalactone. HD methods detected 10 terpenes/terpenoids, whereas 15 were detected through HS‐SPME extraction. Additionally, 13 aromatic compounds were identified in the HS‐SPME extract, whereas none were found in the essential oil. These results highlight the complementary nature of the two extraction methods, as HS‐SPME appears to be more effective in detecting highly volatile compounds that are less efficiently extracted by HD. The two isomers of 2,4‐decadienal were not observed in HS‐SPME. Noteworthy of mention, no compound was detected beyond β‐sesquiphellandrene, which has a molecular weight of 204.19 g/mol. Heavier, less volatile compounds such as farnesenes and oxidation derivatives containing more than 15 carbon atoms were not observed using HS‐SPME.

HD involves harsh conditions, such as high temperatures and water vapor, which can degrade sensitive compounds. In contrast, HS‐SPME operates under milder conditions, providing a more accurate volatile profile that closely reflects the real composition in the organism. HS‐SPME allowed the identification of a greater number of terpenes and aromatic compounds, while HD contains more degradation products; few terpenes, many oxygenated, unsaturated, and fatty acid compounds.

**FIGURE 3:**
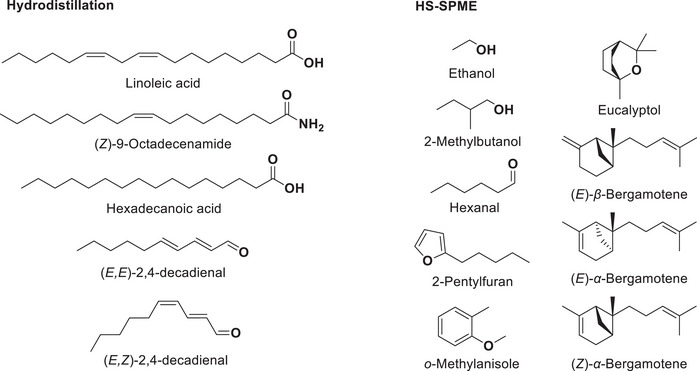
Main volatile compounds identified in the hardwood of *Betula papyrifera* depending of the extraction method: Left. Essential oil obtained by hydrodistillation. Right. Headspace portion obtained by SPME. These compounds are highlighted in bold in Tables 1 and 2. HS‐SPME, headspace solid‐phase microextraction.

**FIGURE 4:**
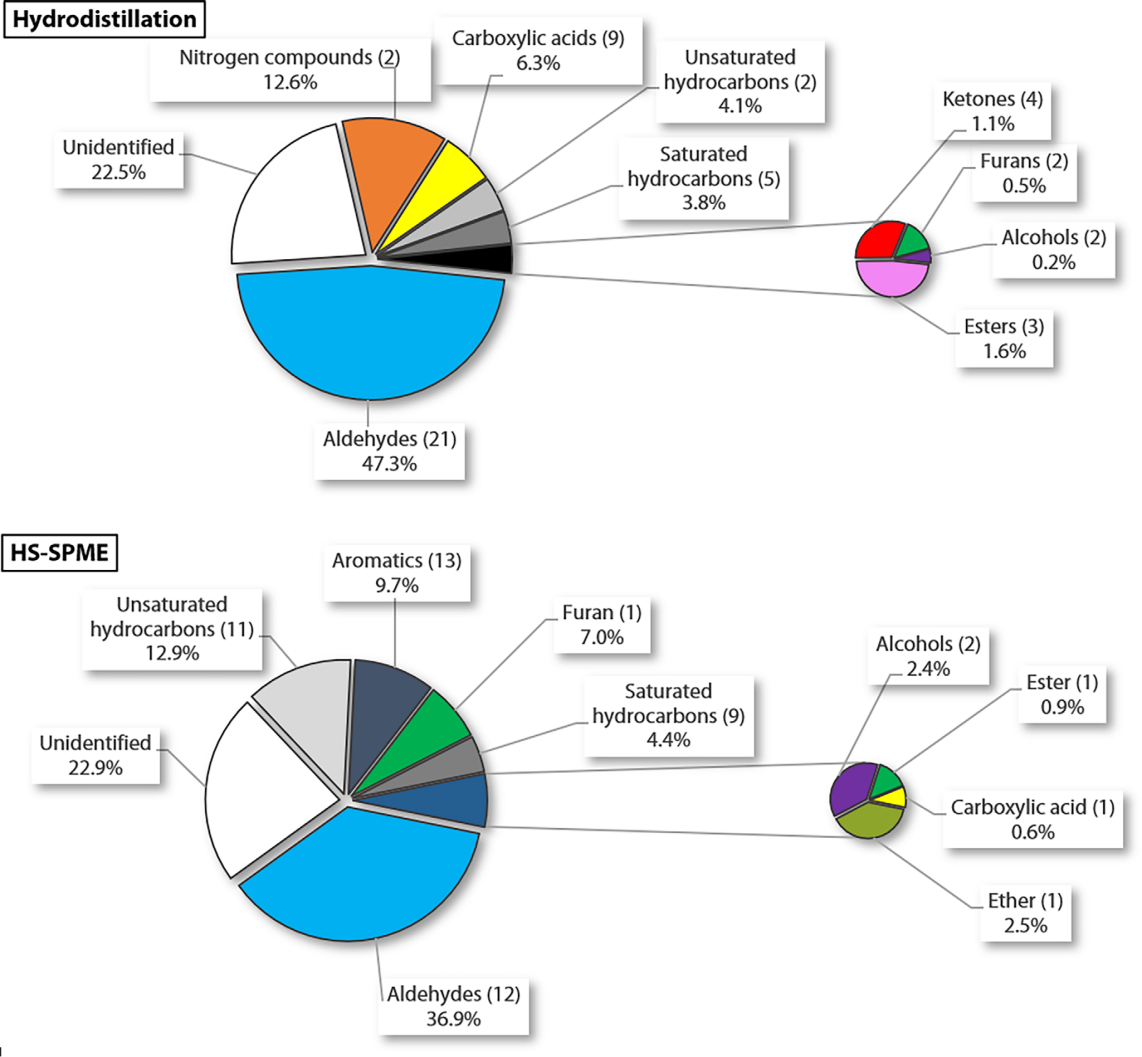
Comparison of the proportions of classes of compounds identified in the hardwood of *Betula papyrifera* depending on the extraction method. Top. Extraction by HD (excluding linoleic acid). Bottom. Headspace extraction by SPME. The number of compounds identified is shown in parentheses. HS‐SPME, headspace solid‐phase microextraction.

Main compounds identified in each extract are shown in Figure 3. Figure 4 compares the proportions of different classes of compounds depending on the extraction method. Linoleic acid, because it makes up most of the essential oil, has been omitted in Diagram A to facilitate comparison. As in the HD extract (excluding linoleic acid), aldehydes (12 compounds) are the dominant class of volatile compounds identified in HS‐SPME. Followed by unsaturated hydrocarbons (11), aromatic compounds (13), a furan (1), and saturated hydrocarbons (9). However, there are considerable differences in the composition of the two extracts.

Aromatic compounds (13) include a variety of structures, such as arenes, oxygenated aromatics, and terpenes. The most abundant aromatic compounds are *o*‐methylanisole (1.8% ± 0.1% to 2.8% ± 0.3%) and *o*‐xylene (0.9% ± 0.3% to 1.7% ± 0.2%). A total of 12 aldehydes were identified in the headspace, hexanal (25% ± 3% to 27% ± 4%) being the most abundant compound, also identified in the essential oil, followed in smaller proportions by 3‐methylbutanal (1.4 ± 0.2 to 1.7% ± 0.6%) and acetaldehyde (1.2% ± 0.4% to 2% ± 1%). Various saturated hydrocarbons (9) were observed in small proportions, with the major compounds being tetradecane (0.64% ± 0.04% to 1.8% ± 0.4%) and dodecane (0.40% ± 0.04% to 1.7% ± 0.3%). The composition of terpenes in headspace portion differs significantly from what is observed in the essential oil and consists mostly of unsaturated hydrocarbons. A total of seven monoterpene compounds were identified, eucalyptol (1.5% ± 0.4% to 4.0% ± 0.5%) and d‐limonene (1.0 ± 0.2% to 1.3 ± 0.2%) being the most predominant. Additionally, compounds **18**, **20**, **21**, and **22** belong to this class, but each exhibits two equally probable identification possibilities that are shown in Table 2. Sesquiterpenoids are more predominant in the headspace extract. Most are unsaturated hydrocarbons, with (*E*)‐α‐bergamotene (2.1% ± 0.2% to 4.8% ± 0.7%) being the most abundant, followed by other isomers such as (*Z*)‐α‐bergamotene (1.2% ± 0.2% to 2.0% ± 0.2%) and (*E*)‐β‐bergamotene (0.7% ± 0.5% to 2.6% ± 0.3%). Other notable compounds include α‐santalene (0.3% ± 0.1% to 0.5% ± 0.2%) and β‐sesquiphellandrene (0.26% ± 0.02% to 0.6% ± 0.2%). None of these compounds were detected in the essential oil of the hardwood in our study, suggesting that the HD process may cause oxidation and degradation of most terpenes [50], or that they are not observed because of their low concentration and the very important proportion of linoleic acid. These sesquiterpenes were previously identified in the *B. papyrifera* inner bark by another group using SPME extraction, with (*E*)‐α‐bergamotene as the major sesquiterpene accompanied by smaller amounts of (*E*)‐ and (*Z*)‐β‐farnesene and (*Z*)‐*α*‐bergamotene, which is consistent with our results [51].

## 3 Conclusions

We characterized the volatile compounds of *B. papyrifera* hardwood using HD and HS‐SPME/GC–MS. HD yielded an essential oil with a low average yield. Its profile is mainly dominated by linoleic acid and lipid oxidation products, suggesting limited potential for direct valorization of the trees through this method. In contrast, HS‐SPME allowed us to identify a different profile as it is more selective toward low molecular weight compounds than HD. This extraction method provided a more representative composition, most probably reflecting the volatile profile of the hardwood itself, as HD appears to cause the loss and degradation of certain compounds. HS‐SPME method revealed additional volatile compounds, including aromatics absent in the essential oil, as well as various terpenes, particularly sesquiterpenoids. Although these results suggest limited viability for extraction of volatile compounds from hardwood alone, future research could explore incorporating bark or using bark exclusively to enhance yields and compound diversity. Combining volatile compound extraction by HD with hot‐water extraction of phenolic compounds could enable a more integrated and sustainable process for the valorization of *B. papyrifera* residues in the forestry industry.

## 4 Experimental Section

### 4.1 Specimens

Three *B. papyrifera* trees were cut in St‐Julien, Quebec, Canada, using a chainsaw on August 3, 2022. The trees were alive and a few dozen meters apart in a mixed Eastern Great Lakes Lowland Forest. For each tree, three pieces at least 29 cm long, with diameters of 11 to 23 cm, were cut at different heights on the tree (bottom, middle, and top). The trees selected for the study are designated Trees 1, 2, and 3; the sampled sections are illustrated in the Supporting Information section. Their ages were determined by dendrochronology, and they are approximately 54, 36, and 83 years old, respectively. Branches and leaves were collected for identification purposes and deposited at the Louis‐Marie Herbarium at Université Laval (Entries # QFA0641221, QFA0641222, QFA0641223).

### 4.2 Sample Preparations

The fresh wood samples were immediately transported to the laboratory in plastic bags and stored in the freezer at −20°C. The bark was then removed, and the wood was cut into strips approximately 2.5 cm in diameter. We only kept the pieces in good condition, without visible blemishes (bark, rot, or coloring), for analysis. We then chipped the strips with a Yardworks wood shredder machine. The chips were stored at −20°C in plastic bags until they were analyzed. More details on the process can be found in the Supporting Information section. The wood chips, obtained from the three pieces of each individual tree, were evenly combined and labeled separately for each of the three trees. These steps were all carried within 24 h after cutting the trees.

### 4.3 Essential Oils Extraction by HD

The volatile compounds from the hardwood of *B. papyrifera* were extracted within 1 month of tree cutting using HD with a Clevenger‐type apparatus. In the process, 500 g of fresh wood chips were added to a 5 L flask containing 2.5 L of deionized water and heated to boiling for 5 h. The essential oil was collected by adding three portions of 5 mL diethyl ether to the Clevenger apparatus. The oil was then dried with Na_2_SO_4_, followed by filtration and evaporation under vacuum at room temperature using a rotary evaporator. A yellowish oil with a grass and chicken stock smell was obtained. The samples obtained were stored in glass vials at 4°C and protected from light until analysis. The essential oil yield (% w/w) was calculated from the dry mass.

### 4.4 Analysis of the Volatile Compounds Obtained by HD Using GC–MS and GC–FID

Volatile compounds in the samples were separated and identified using a Thermo Scientific GC–MS system (Trace GC Ultra with DSQ II detector) with a DB‐5MS non‐polar phase column (30 m × 0.25 mm × 0.25 µm). The carrier gas was helium flowed at a constant rate of 1 mL/min, a split ratio of 10, and an injector temperature of 240°C. The mass range was 40–400 Da, and the ionization energy was set at 70 eV, controlled by Xcalibur 4.0 software. The essential oil was diluted to 10% w/w in dichloromethane, then a 1.0 µL volume was injected in the instrument. The temperature program was set as follows: 50°C for 5 min, 2°C/min increase to 200°C, then to 270°C at 10°C/min, held at this temperature for 5 min.

We used Thermo Scientific's QualBrowser software to process the data and identified compounds by comparing their mass spectra and linear retention indices (LRIs) to the commercial spectral libraries FFNSC 3 from Wiley (Flavor and Fragrance Natural and Synthetic Compounds) [35] and NIST 14 (National Institute of Standards and Technology) [34].

The standard solution for the determination of LRIs was prepared from a solution of C7 to C30 alkanes (Millipore Sigma, 49451‐U) diluted to 200 µg/mL in dichloromethane. The LRIs were then calculated using the Van den Dool and Kratz equations [52]. GC/FID data were used to calculate relative proportions. The percentage of each compound in the total extracts is expressed as the ratio of the area of each individual signal to the total integration area of the chromatogram. We manually integrated the signals without any correction factors. Each of the nine essential oils obtained was subject to a single analysis for identification purposes, and then to triplicate analyses in GC/FID for semi‐quantification. The results are expressed as relative proportions (% ± standard deviation) of volatile compounds grouped for all essential oils from the same tree.

The GC–FID used was a Thermo Trace GC Ultra (Thermo Scientific) with a Triplus autosampler. The column used, the injection conditions, and the temperature ramp are the same as those specified for GC/MS analyses. The FID temperature was 300°C, and gas flow rates were as follows: air = 350 mL/min; H_2_ = 35 mL/min; N_2_ = 30 mL/min.

### 4.5 Extraction of Volatile Compounds by Headspace Solid‐Phase Microextraction

We extracted the volatile compounds using an SPME fiber composed of a 50/30 µm layer of DVB/CAR/PDMS (Supelco 57328‐U). The SPME fiber was conditioned before analysis according to the supplier's instructions. We ran a blank to check the cleanliness of the fiber and observed signals associated with siloxanes in all analyses; we therefore ignored them during integration. Approximately 2 g of fresh wood chips were introduced into a 20 mL SPME vial closed by a cap fitted with a silicone/PTFE septum. The vial was immersed in a water bath at 65°C for 5 min to allow headspace compounds to reach equilibrium. The fiber was then introduced and exposed to the headspace for 30 min. The compounds were finally desorbed manually in the injection port of the GC–MS. Each analysis was completed in triplicate to account for instrumental variability.

### 4.6 Analysis of Headspace Volatile Compounds by GC–MS

The volatile compounds were separated and analyzed using the same instrument, carrier gas, flow rate, ionization energy, and column employed in the analysis of the essential oils obtained by HD with GC–MS.

Thermal desorption of the compounds was carried out in the GC injector at 230°C for 5 min in splitless mode. The temperature program was set as follows: 40°C for 5 min, then an increase of 3°C/min to 140°C, then to 220°C at 12°C/min, and 5 more minutes at the final temperature. The mass range was 40–350 Da. The identification of the compounds was carried out with the same software and using the same method as described above for the essential oil obtained by HD.

The percentage of each compound in the total extraction product is expressed by the ratio of the area of each individual signal to the total integration area of the chromatogram. The signals were integrated manually and without the use of correction factors. Three analyses were conducted per tree for the wood chips of each of the three samples to illuminate aspects of both instrumental and intra‐tree variability. All analyses were completed within a week of the harvest.

## Author Contributions

David Fortier did the sample collection, laboratory work, and analyses and wrote the first draft. Jean‐Christophe Séguin performed the preliminary work on the project, contributed to the conception of the project, and reviewed the manuscript. Normand Voyer conceived the project and research plan, supervised the lab work, obtained financial support, and reviewed the manuscript.

## Conflicts of Interest

The authors declare no conflict of interest

## Supporting information




**Supporting file 1**: cbdv70230‐sup‐0001‐SupMat.docx

## Data Availability

Data Availability Statement The data that support the findings of the study are openly available in Borealis at https://doi.org/10.5683/SP3/2RDTAJ.
