# Supplementary material for: Characterization of the Volatile Compounds of the Hardwood Portion of Betula papyrifera Marshall From Quebec, Canada
Source: Chem Biodivers. 2025 Jul 18;22(11):e01398. doi: 10.1002/cbdv.202501398 (PMC12629156; doi:10.1002/cbdv.202501398)
Supplement: Supplementary file 1 — Supporting file 1: cbdv70230‐sup‐0001‐SupMat.docx [file CBDV-22-e01398-s001.docx]

## Supporting information

## Table of Contents

### Preparation of Wood Samples

- **Figure S1.** Samples from Trees 1, 2, and 3 of paper birch (*Betula papyrifera*) from 3 live trees harvested in the forest near St-Julien on August 3, 2022.
- **Figure S2**. (a) Process for reducing wood samples into strips approximately 2.5 cm in diameter. (b) Selection of hardwood strips without bark or visible alterations.
- **Figure S3.** Yardworks wood shredder machine used to reduce wood strips into woodchips.

### Chromatograms of the extracts of *B. papyrifera*

- **Figure S4.** Chromatograms obtained by HS-SPME/GC-MS analysis of the hardwood of 3 *B. papyrifera* trees collected in St-Julien, Quebec, Canada.
- **Figure S5.** Chromatograms obtained by GC-MS analysis of the volatile extract obtained by hydrodistillation on the hardwood of 3 *B. papyrifera* trees collected in St-Julien, Quebec, Canada.
- **Figure S6.** Chromatograms obtained by GC-FID analysis of the volatile extract obtained by hydrodistillation on the hardwood of 3 *B. papyrifera* trees collected in St-Julien, Quebec, Canada. Legend key: The first digit represents the extract number, while the second digit indicates the replicate.

## Preparation of Wood Samples


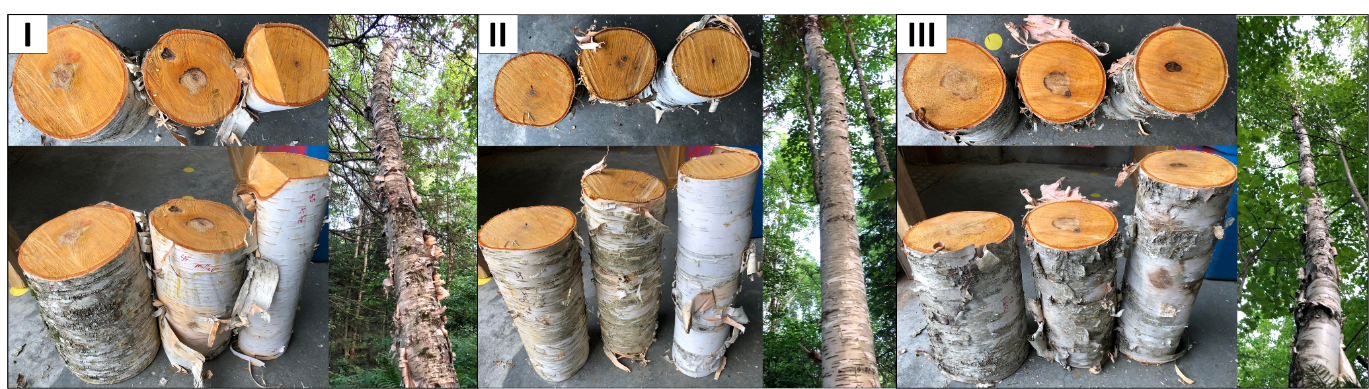


**Figure S1.** Sampling 3 live paper birch (*B. papyrifera*) trees from in the forest near St-Julien on August 3, 2022.

**
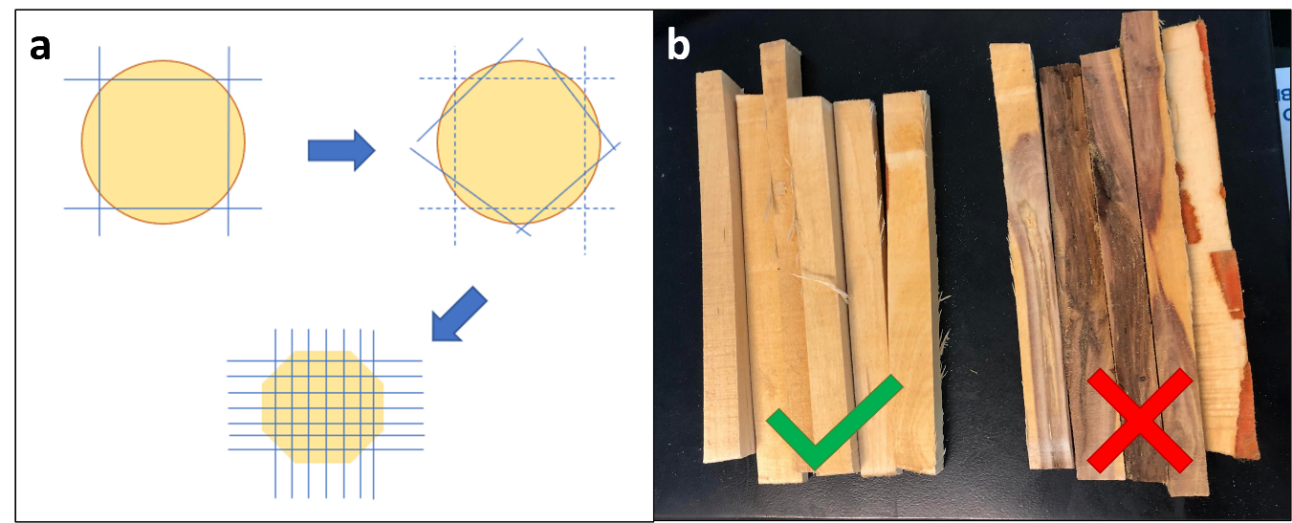
**

**Figure S2.** (a) Process for reducing wood samples into strips approximately 1 inch in diameter. (b) Selection of hardwood strips without bark or visible alterations.


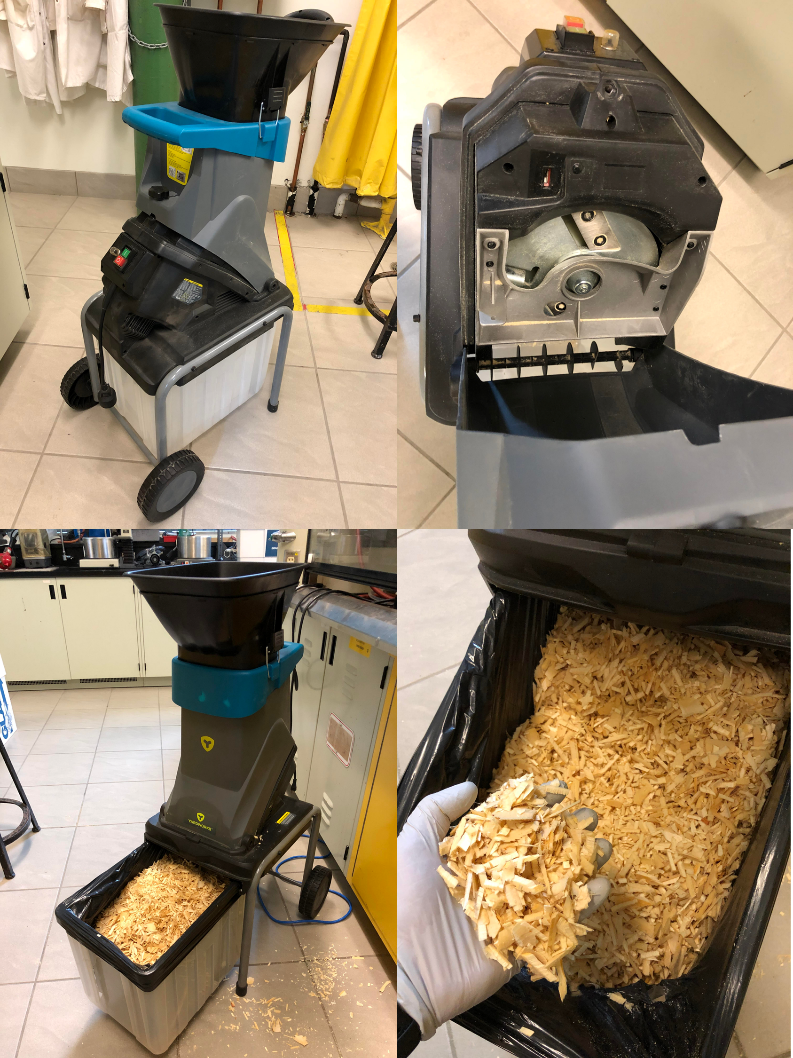


**Figure S3.** Yardworks wood shredder machine used to reduce wood strips into woodchips.

## Chromatograms of the extracts of *B. papyrifera*


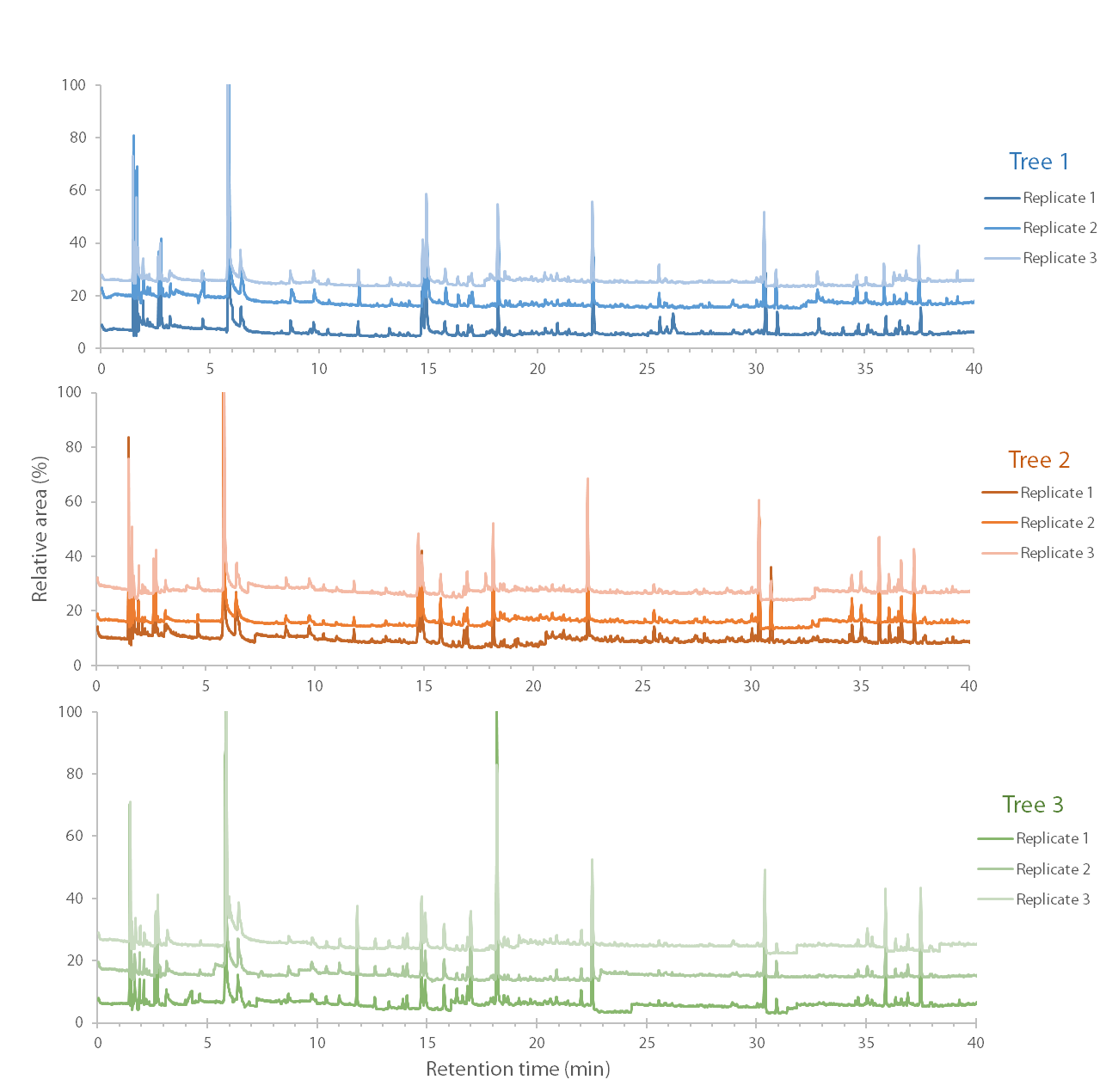


**Figure S4.** Chromatograms obtained by HS-SPME/GC-MS analysis of the hardwood of 3 *B. papyrifera* trees collected in St-Julien, Quebec, Canada.


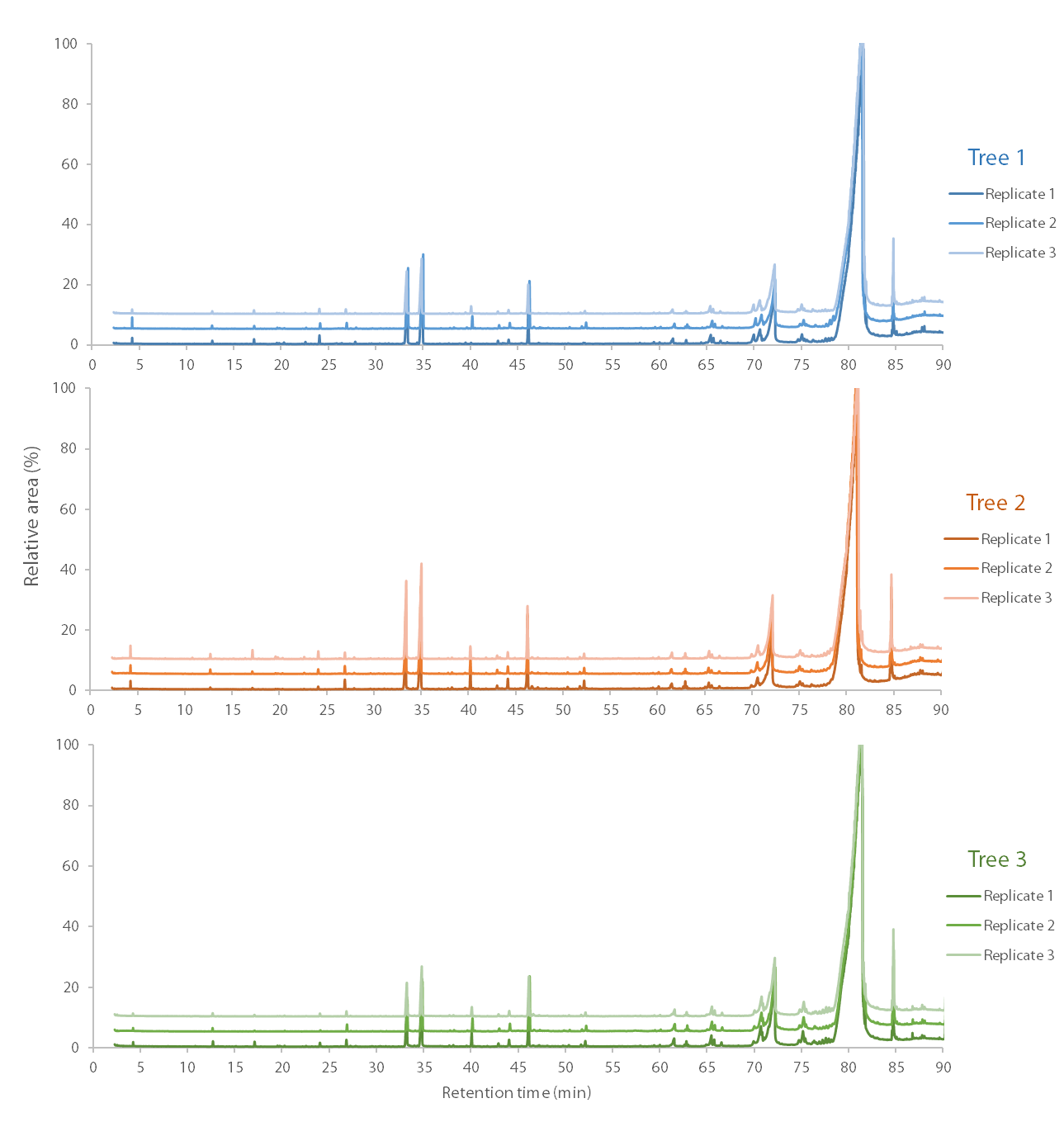


**Figure S5.** Chromatograms obtained by GC-MS analysis of the volatile extracts obtained by hydrodistillation on the hardwood of 3 *B. papyrifera* trees collected in St-Julien, Quebec, Canada.


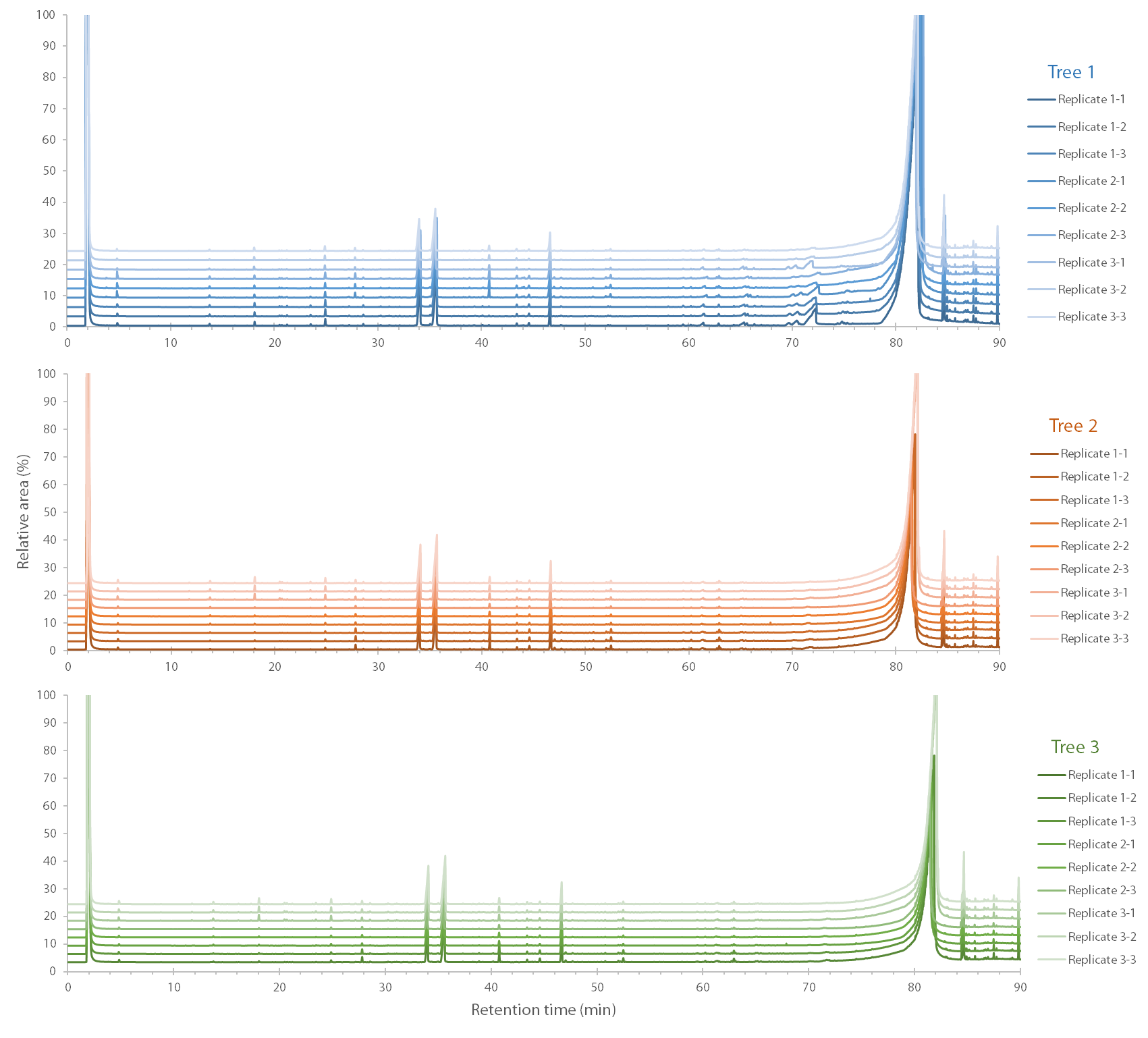


**Figure S6.** Chromatograms obtained by GC-FID analysis of the volatile extract obtained by hydrodistillation on the hardwood of 3 *B. papyrifera* trees collected in St-Julien, Quebec, Canada. Legend key: The first digit represents the extract number, while the second digit indicates the replicate.
